# Study of Process, Microstructure, and Properties of Double-Wire Narrow-Gap Gas Metal Arc Welding Low-Alloy Steel

**DOI:** 10.3390/ma17246183

**Published:** 2024-12-18

**Authors:** Ning Xiao, Haoyu Kong, Qingjie Sun, Ninshu Ma

**Affiliations:** 1 METALLECO Inc., No. 3185 Dundas St. W, 2F, Oakville, ON L6M 4J4, Canada; nick.xiao@metalleco.ca; 2 Joining and Welding Research Institute, Osaka University, Osaka 567-0047, Japan; 3 STUAA Automation Co., Ltd., No. 1000 South Langyatai Road, Qingdao 266400, China; 15166703946@163.com

**Keywords:** double-wire narrow-gap welding, welding parameters, heat input, microstructure, microhardness, numerical simulation

## Abstract

Narrow-gap arc welding is an efficient method that significantly enhances industrial production efficiency and reduces costs. This study investigates the application of low-alloy steel wire EG70-G in narrow-gap gas metal arc welding (GMAW) on thick plates. Experimental observations were made to examine the arc behavior, droplet transition behavior, and weld formation characteristics of double-wire welding under various process parameters. Additionally, the temperature field of the welding process was simulated using finite element software (ABAQUS 2020). Finally, the microstructure and microhardness of the fusion zone in a double-wire, single-pass filled joint under the different welding speeds were compared and analyzed. The results demonstrate that the use of double-wire GMAW in narrow-gap welding yielded positive outcomes. Optimal settings for wire feeding speed, welding speed, and double-wire lateral spacing significantly enhanced welding quality, effectively preventing side wall non-fusion and poor weld profiles in the welded joints. The microstructure of the fusion zone produced at a higher welding speed (11 mm/s) was finer, resulting in increased microhardness compared to welds obtained at a lower speed (8 mm/s). This is attributed to the shorter duration of the liquid molten pool and the faster cooling rate associated with higher welding speed. This research provides a reference for the practical application of double-wire narrow-gap gas metal arc welding technology.

## 1. Introduction

In recent years, advancements in heavy machinery, marine engineering equipment, and pressure vessel manufacturing have led to a trend of increasing equipment size. Thick plates are the primary raw materials for the construction of such equipment, and welding is a critical process in their manufacturing [1,2]. Traditional thick-plate structures typically employ techniques such as multi-pass welding with large grooves or submerged arc welding [3]. However, the large groove dimensions and significant metal fill requirements associated with these methods lead to a substantial increase in the volume of welding work. This, in turn, limits production efficiency and raises welding costs. Additionally, the considerable metal fill and multi-pass heat input during thick-plate welding can adversely affect the mechanical properties of the welded joints and can cause significant residual stress and deformation [4]. This will significantly undermine joint quality and adversely affect the service performance of the welded structures.

Narrow-gap welding technology has emerged as an effective method for achieving high-quality welding in thick plates [5,6]. This technique has advantages such as smaller groove dimensions and reducing the amount of filling metal required. Consequently, this leads to a marked decrease in welding workload and production costs [7]. Furthermore, the reduced heat input and higher cooling rates associated with narrow-gap welding effectively mitigate residual stress and deformation in the welded joints. As a result, narrow-gap welding is recognized as a high-efficiency, high-quality welding technique, establishing its dominant position in the manufacturing of thick-walled structures [8].

Since the introduction of narrow-gap welding technology, numerous researchers have explored various aspects of the technique. Current research predominantly focuses on narrow-gap GMAW [9,10], narrow-gap tungsten inert gas (TIG) [11,12] welding, and narrow-gap laser welding [5,13], which have made great progress. Despite its efficiency, lack of fusion on side walls remains the most common defect that restricts the ability to achieve high-quality welding [14]. To address this issue, some researchers have introduced the oscillation of the heat source or the welding wire during the welding process to effectively heat the side wall metal, thus achieving a proper metallurgical bond [15,16,17]. Moreover, some researchers have employed hybrid welding approaches that incorporate multiple heat sources into the narrow-gap welding process, effectively leveraging the advantages of various techniques to achieve high-quality welding [18,19].

Narrow-gap GMAW, with its high welding efficiency and low cost, is widely used in the manufacturing of thick-walled steel structures [20]. To further enhance welding efficiency and reduce the likelihood of defects, various techniques based on GMAW, including oscillating GMAW [21], cable-type wire GMAW [22], and multi-wire GMAW [23,24], have attracted considerable attention. Among these, double-wire GMAW has gained traction due to its capacity for independent adjustment of both welding wire parameters, providing a broader processing window and greater flexibility [25]. This technique has been utilized in various sectors [26,27,28], and the process characteristics of double-wire welding have also been studied. Han et al. [29] studied the effects of double-wire angle on the fluid dynamics and weld defects in the narrow-gap GMAW of 5083 Al-Mg alloy through experimental and simulation methods. They found that an increase in the interwire angle proves beneficial for reducing porosity and improving weld formation; however, excessive interwire angles (14°) can lead to increased porosity in the sidewall region. Liu et al. [23] studied the effects of double-wire angle on the welding process stability and arc behavior during narrow-gap welding. They found that increasing the interwire angle to a certain value could improve the welding process stability during double-wire narrow-gap GMAW. Cai et al. [30] studied the effects of shielding gas composition on weld seam appearance during double-wire narrow-gap GMAW. They found that the weld width was the largest when using the mixture of 80% Ar + 10% CO_2_ + 10% He as the shielding gas. P.H.G. et al. [31] performed the narrow-gap welding of thick-plate high-strength low-alloy steel pipe through double-wire GMAW. They found that an increase in the inter-pass temperature reduced the microhardness and impact toughness of weld metal.

Most of the current research focuses on the influence of a single parameter on welding. This research aims to investigate double-wire GMAW for the narrow-gap welding of low-alloy steel, systematically studying the welding process, weld formation, microstructure, and microhardness, and establishing the influence relationship between them. The findings of this study will enrich the understanding of narrow-gap welding processes and promote the practical application of double-wire narrow-gap GMAW in industrial settings, contributing to advancements in welding technology and practices.

## 2. Materials and Methods

**Figure 1:**
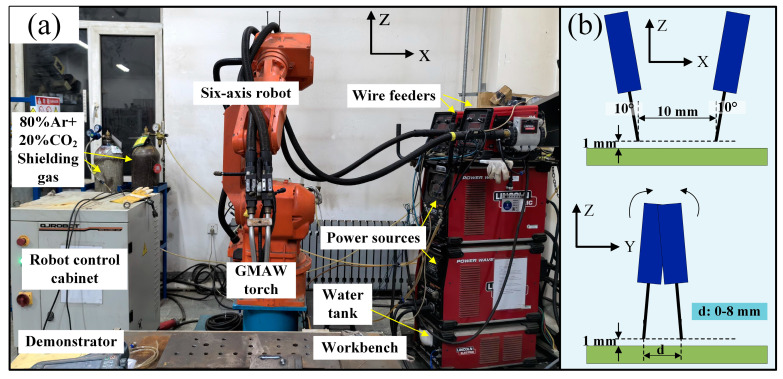
(**a**) Double-wire narrow-gap GMAW system and (**b**) arrangement of the double wire.

To achieve the high-efficiency narrow-gap welding of thick-walled structures, a double-wire GMAW system was employed in this study, as depicted in Figure 1a. The welding system consisted of a six-axis robot, a double-wire GMAW torch, two welding power sources, two wire feeders, and two bottles of shielding gas of 80% Ar and 20% CO_2_. It is worth noting that the double-wire welding torch used in this study is composed of two single-wire welding torches. Two welding wires pass through these two single-wire welding torches, with process parameters controlled by two welding power sources. Constant voltage mode was used during welding, and both welding wires were melted simultaneously. The gas flow rate for both welding torches was set to 20 L/min to obtain a good protection effect. The hybrid torch was fixed on the six-axis robot to achieve the movement. The welding direction was along the X-axis. Figure 1b shows the projection of two wires on the ZX plane and ZY plane, clearly indicating the arrangement of the double wire. Along the welding direction, the two wires were arranged in front of one another, and they were tilted with an angle of 10° from the vertical direction. The distance between the ends of the two wires was 10 mm (under the condition of wire extension of 25 mm), and the distance between the ends of the welding wires and the substrate was 1 mm. It is worth noting that the two wires were flexible in the direction perpendicular to the welding direction. The wires could be rotated around the *X*-axis, and the distance between the ends of the two wires varied from 0 mm to 8 mm (under the condition of constant wire extension of 25 mm).

The base material used in the welding processes was Q235, and the welding wire was ER70-G. The chemical compositions of the materials are shown in Table 1.

**Figure 2:**
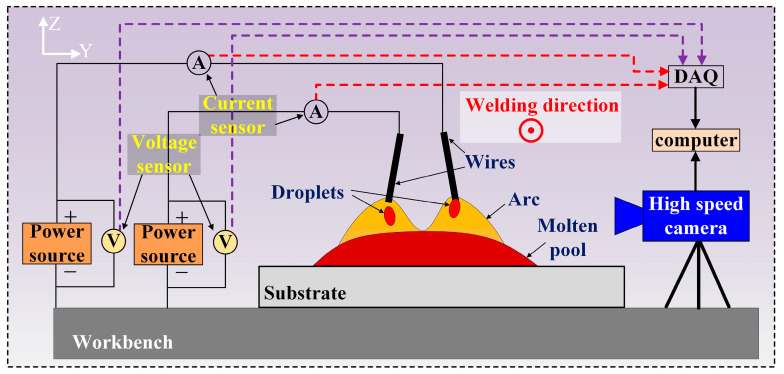
Schematic diagram of welding process on plates.

In order to obtain suitable process parameters for narrow-gap welding, welding was first conducted on the Q235 substrate with a thickness of 10 mm to study the arc shape, droplet transition mode, and current–voltage waveforms. Firstly, welding experiments were conducted on a flat plate through single-wire welding using the parameters shown in Table 2, and the welding voltage was automatically matched with that of the welding power source. Then, double-wire welding was conducted using the optimized wire feeding speed. As shown in Figure 2, a high-speed camera (OLYMPUS I-SPEED, Shanghai, China) was used to capture the behavior of the arc and droplet transfer during the welding process. The camera was placed perpendicularly to the welding direction, and the camera frame rate and exposure time were 2000 fps and 2 μs, respectively. Simultaneously, an electrical signal acquisition device was used to collect the current–voltage waveforms of the arc during welding. The electrical signal data of two arcs were collected simultaneously, with a sampling frequency of 1000 Hz. The information collected during the welding process was processed and analyzed to study the stability of the welding process and the interaction behavior between the double arcs.

**Figure 3:**
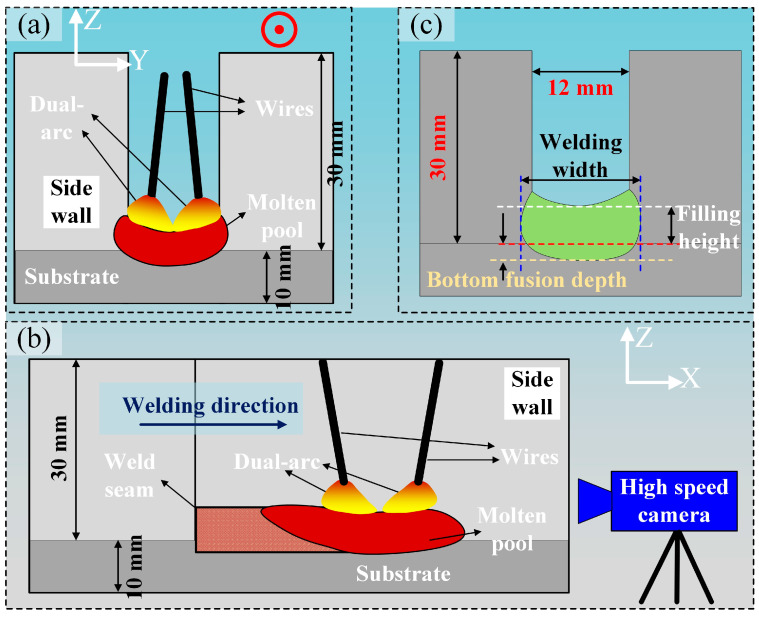
Schematic diagram of (**a**,**b**) welding process in the narrow-gap groove and (**c**) measurement of the weld bead.

Subsequently, narrow-gap groove filling was conducted using the double-wire welding process. The welded assembly comprised a narrow-gap groove with a width of 12 mm, which was created by joining two 30 mm thick steel plates as sidewalls with a 10 mm thick steel plate as substrate, as illustrated in Figure 3a,c. During the welding, changes were made to parameters such as the welding speed and double-wire lateral spacing at the wire feeding speed of 8 m/min for the front and back wires. The detailed parameters are shown in Table 3. The arc and droplet transition behavior under different double-wire lateral spacing were photographed, as shown in Figure 3b. After single-pass filling welding was completed, metallographic samples were taken using wire electrical discharge, and the samples’ cross-section was polished sequentially with 80# and 400# sandpaper and then etched with a nitric acid–alcohol solution. Data on various factors such as the welding width, filling height, and bottom fusion depth from the cross-section morphology of the weld bead were extracted to study the influence of the welding process on the formation of weld seams and defects.

Finally, a comparative analysis of the microstructure and mechanical properties of the weld joints obtained at different welding speeds was conducted. Metallographic samples were cut from the welded structure using wire cutting, and the surfaces were polished with 80#–1500# sandpaper, followed by diamond polishing paste with a diameter of 2.5 μm, and then etched with a nitric acid–alcohol solution for 20 s. Metallographic images were then captured using an optical microscope (OLYMPUS DSX510, Beijing, China). Subsequently, microhardness testing was performed using a Vickers hardness tester (HVS-1000, HST Group, Aurora, IL, USA) with a load of 200 g and a loading time of 15 s.

## 3. Results and Discussion

### 3.1. Dynamic Behaviors of Arc and Droplets

**Figure 4:**
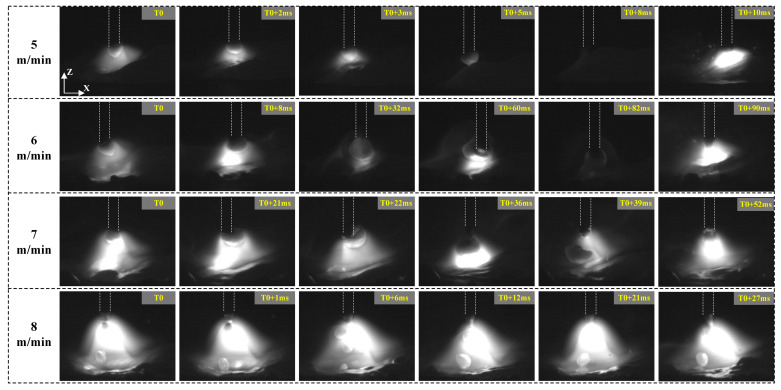
Arc and droplet behaviors under different wire feeding speeds (5 m/min, 6 m/min, 7 m/min, and 8 m/min) from side view.

**Figure 5:**
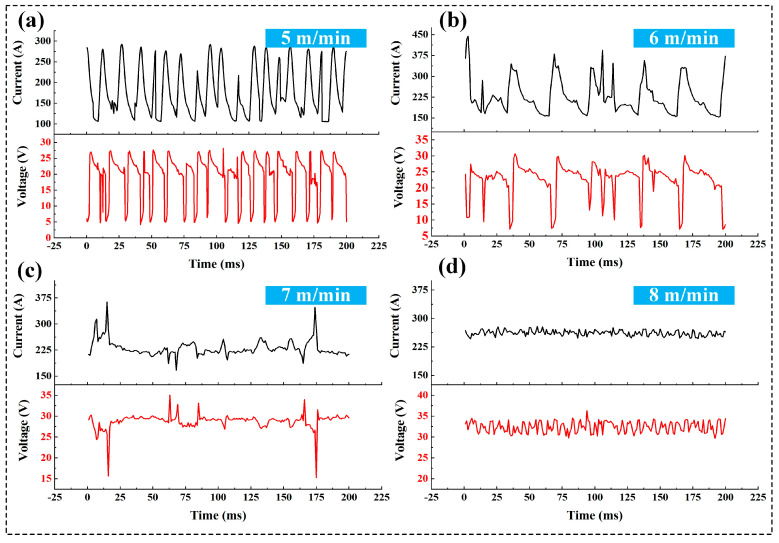
Current–voltage waveforms under different wire feeding speeds: (**a**) 5 m/min; (**b**) 6 m/min; (**c**) 7 m/min; (**d**) 8 m/min.

The photos captured under different wire feeding speeds are shown in Figure 4. It can be observed that as the wire feeding speed increases, the arc becomes larger and brighter. The corresponding current–voltage waveforms in Figure 5 indicate that as the wire feeding speed increases, both the welding current and voltage increase.

At a wire feeding speed of 5 m/min, the wire end melts, and the droplet grows continuously. Due to the lower voltage, when the droplet grows to a certain size and touches the molten pool, the arc diminishes. At this moment, the voltage is at a minimum, while the current is at a maximum. Subsequently, the arc spikes again, the voltage increases, and the current decreases (shown in Figure 5a). The droplet at the wire end transitions to the molten pool in this periodic short-circuit manner. The increased wire feeding speed to 6 m/min maintains the short-circuit transition mode for the droplet. However, due to the higher voltage at this speed compared to that of 5 m/min, the droplet grows continuously before contacting the molten pool, with a larger volume than at 5 m/min. Moreover, the droplet at the wire end receives resistance from the anode spot, hindering its transition behavior, which also results in a larger droplet size and increased transition periods. The current–voltage waveforms in Figure 5b clearly show the elongation of the droplet transition period. When the wire feeding speed increases to 7 m/min, the welding current and voltage further increase, causing the wire end to melt and grow. Although the droplet is still hindered by the anode spot, when it reaches a sufficient size, it detaches from the wire end and transitions to the molten pool. However, Figure 5c shows that the droplet transition behavior at this speed was unstable. When the wire feeding speed reaches 8 m/min, the welding current and voltage are high enough for the wire to melt significantly, resulting in a pointed shape at the wire end, and the droplet with a diameter close to the diameter of the wire transitions to the weld pool in a spray-like manner. Figure 5d demonstrates stable current–voltage waveforms, indicating a consistent spray transition mode at this parameter.

**Figure 6:**
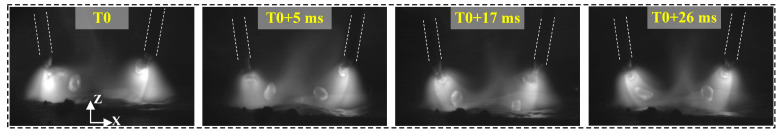
The behaviors of double-wire arcs and droplets at a wire feeding speed of 8 m/min from side view.

In order to ensure the successful progress of the narrow-gap welding, sufficient heat input must be provided to fill the narrow-gap groove without causing the defect of lack of fusion on the sidewalls. It is also necessary to adopt stable welding process parameters to ensure the stability of the welding operation. Therefore, the wire feeding speed of 8 m/min seems to be a very suitable process parameter. Subsequently, a welding experiment was conducted on the Q235 plate using double-wire welding. Both the wires were at a wire feeding speed of 8 m/min, and the welding voltage was automatically set to 32.5 V using the welding power source. Figure 6 shows the transition behavior of the arcs and droplets during the double-wire welding process. At a wire feeding speed of 8 m/min, the droplets from the two wires transitioned to the molten pool in a spray manner. Moreover, the two wires melted, thus forming a common molten pool.

**Figure 7:**
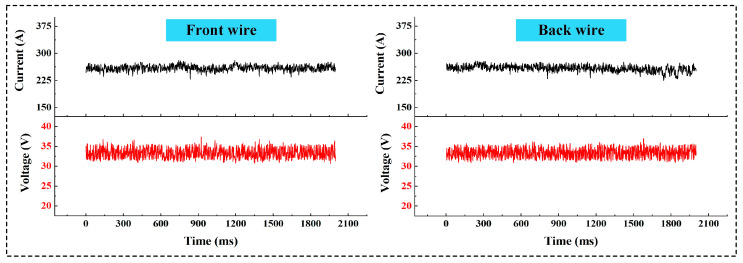
Current–voltage waveforms during the double-wire welding process.

In the double-wire welding process, the interference between the two arcs is the main factor affecting the stability of the welding process [28]. However, in this study, no impact on welding stability was observed, as evidenced by the current–voltage waveforms shown in Figure 7. The stability of the double-wire welding process may be attributed to the self-regulating function of the welding power source.

**Figure 8:**
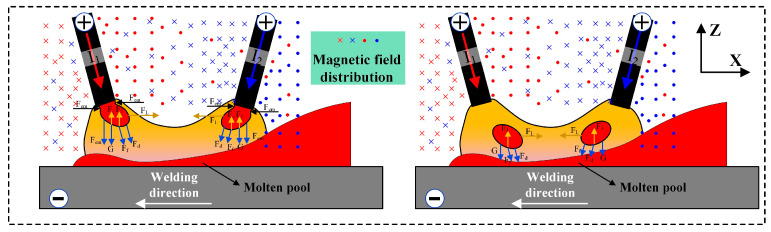
Force analysis of the droplet transition during the double-wire welding process.

It is worth noting that during the double-wire welding process, the two arcs attract each other, resulting in a noticeable arc deflection. Additionally, the droplets exhibit a movement toward the center between the two arcs. This is the result of the interaction of magnetic fields produced by the two wires. During welding, the welding power sources are connected in a circuit with the wires and the workbench, with the current flowing from the wires through the arc to the workbench. Figure 8 illustrates the behavior of the arcs and droplets in the magnetic field during the double-wire welding process. The red and blue symbols in the figure represent the magnetic field distribution of the front and back wires, respectively. Additionally, the cross marks symbolize the direction perpendicular to the page inward, and the dot marks symbolize the direction perpendicular to the page outward. The plasma is ultimately deflected due to the action of the Lorentz force. The droplets detached from the end of the wires under the action of the electromagnetic pinch force (F_em_), plasma flow force (F_d_), gravity (G), frictional force (F_f_), surface tension (F_γ_), metal vapor recoil force (F_J_), and Lorentz force (F_L_) [32,33]. As the resultant force on the droplets does not align with the axis of the welding wire, the droplets ultimately transition toward the center of the molten pool.

### 3.2. Weld Bead Profile and Formation Mechanism

**Figure 9:**
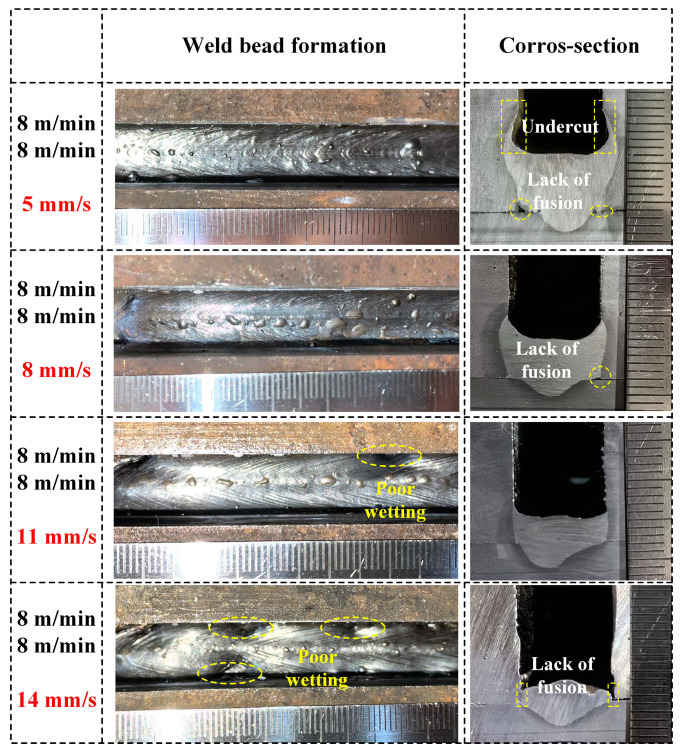
Weld bead formation and cross-section under different welding speeds (5 mm/s, 8 mm/s, 11 mm/s, and 14 mm/s) at a wire feeding speed of 8 m/min for the double wires.

**Figure 10:**
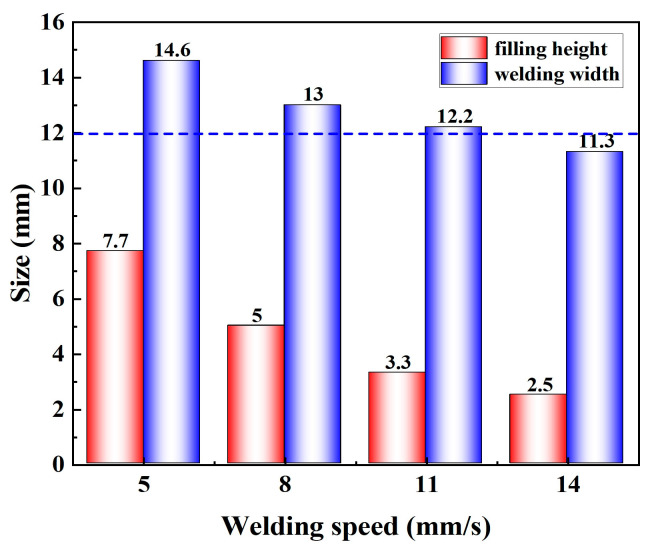
Measurement results of the weld bead section.

Using the process parameters described above for narrow-gap welding, a wire feeding speed of 8 m/min was adopted for the two wires in the subsequent welding process. Initially, experiments were conducted without a double-wire lateral distance, meaning the two wires were in the same plane. Figure 9 displays the weld seam formation and cross-sectional morphology under different welding speeds. It is clear from an overhead view that as welding speed increases, the scale-like patterns on the weld seam surface become denser. At welding speeds of 5 mm/s and 8 mm/s, the weld seam surface appears aesthetically pleasing. However, at higher welding speeds of 11 mm/s and 14 mm/s, insufficient heat input leads to the poor wettability of the filling metal on the sidewalls. The cross-sectional morphology clearly illustrates the weld seam. At a welding speed of 5 mm/s, with higher heat input and more filling metal deposition, an undercut is observed, and the defect of lack of fusion at the groove root corner occurs. Decreasing heat input may contribute to suppressing undercut defects, but inadequate heat input will lead to the defect of lack of fusion on the sidewalls. Measuring was conducted on the filling height and welding width of the weld seam, with the results shown in Figure 10. As the welding speed increases from 5 mm/s to 14 mm/s, the filling height of the weld seam is reduced from 7.7 mm to 2.5 mm, and the welding width is reduced from 14.6 mm to 11.3 mm.

Although undercut defects and lack of fusion on the sidewalls can be avoided at welding speeds of 8 mm/s and 11 mm/s, lack of fusion at the groove root corner and poor sidewall wettability may occur. This may result from the two wires being in the same plane, causing heat concentration at the center of the groove. If the two wires are angled laterally with each wire pointing toward a side wall of the groove, the temperature field distribution within the groove may be improved, potentially improving weld seam formation. The welding torch was then adjusted so that the two wires would have a certain lateral spacing. Experiments were conducted with different lateral distances between the two wires at welding speeds of 8 mm/s and 11 mm/s.

**Figure 11:**
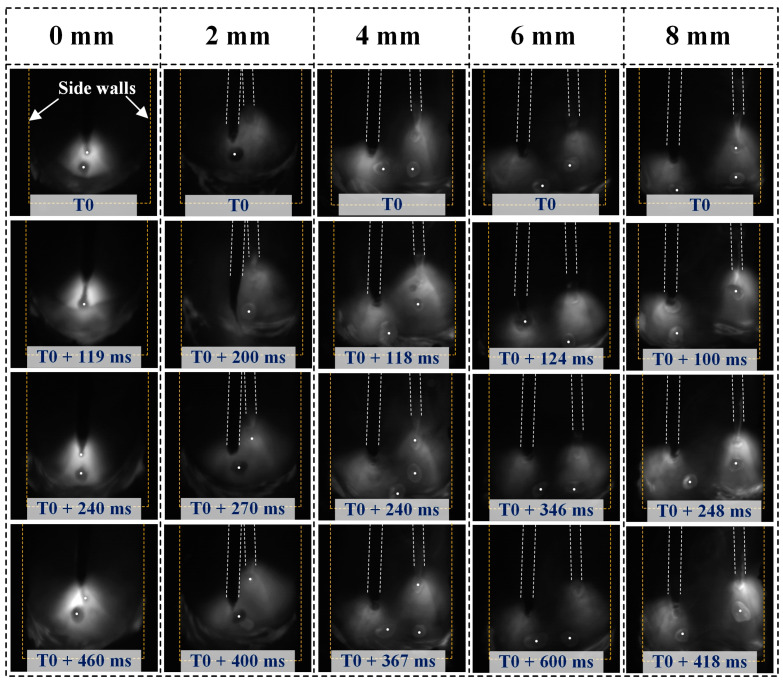
Arc and droplet behavior under different lateral spacings (0 mm, 2 mm, 4 mm, 6 mm, and 8 mm) at a welding speed of 11 mm/s from the front view.

The images captured with a high-speed camera, as illustrated in Figure 11, display the behaviors of the arc and droplets under varying lateral distances at a welding speed of 11 mm/s. At a lateral spacing of 0 mm between the double wires, the arc is confined to a small area within the narrow-gap groove, primarily concentrated in the center. The droplets transition to the molten pool via a spray transition method. As the lateral spacing of the double wires increases, the arc’s influence expands gradually from the center of the groove to encompass the entire groove, resulting in varying distributions of the temperature field. When the spacing reaches 8 mm, the arc begins to interact with the side walls of the narrow-gap groove. Notably, the mode of droplet transition remains unchanged across different lateral spacings, consistently exhibiting a spray transition. It is evident that the droplets do not migrate to the molten pool along the wires; instead, they show a tendency to move toward the center of the molten pool.

**Figure 12:**
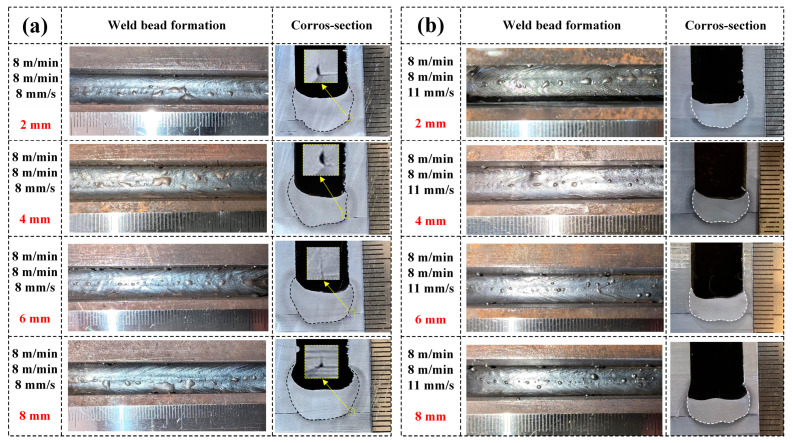
Weld bead formation and cross-section under different lateral spacings (2 mm, 4 mm, 6 mm, and 8 mm) at a wire feeding speed of 8 m/min for the double wires and welding speeds of (**a**) 8 mm/s and (**b**) 11 mm/s.

**Figure 13:**
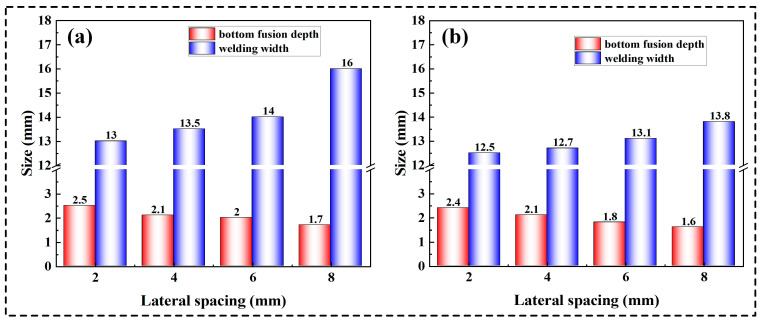
Measurement results of the weld bead section at the welding speed of (**a**) 8 mm/s and (**b**) 11 mm/s.

Figure 12a,b illustrate the weld seam formation and cross-sectional morphology obtained under different lateral wire distances at welding speeds of 8 mm/s and 11 mm/s, respectively. It can be seen that with the presence of an angle between the two wires, the weld seam is well formed. When the welding speed is 11 mm/s, there is no poor wetting condition observed. Compared to the situation where the two wires are not angled, the presence of lateral spacing between the two wires effectively improves the weld seam formation, specifically manifested in an improvement in the weld seam cross-sectional morphology. The presence of lateral spacing between the two wires will effectively improve the temperature field inside the narrow-gap groove, allowing more heat to be utilized on both sides of the groove. As the lateral spacing between the two wires increases, the weld seam cross-section gradually becomes more rounded, and the shape of the fusion depth at the bottom of the groove changes from finger-like to bowl-shaped. Additionally, as the lateral spacing between the two wires increases, the welding width of the weld seam increases, and the bottom fusion depth decreases. However, when the lateral spacing between the two wires becomes too large, it can lead to undercut defects, a phenomenon observed at welding speeds of both 8 mm/s and 11 mm/s. Figure 13a,b show the specific information on the weld seam size under different lateral spacing conditions at welding speeds of 8 mm/s and 11 mm/s, respectively, which clearly demonstrate regular shapes. When the welding speed is 8 mm/s, the bottom fusion depth of the weld seam decreases from 2.5 mm to 1.7 mm, and the welding width increases from 13 mm to 16 mm with the increase in spacing. When the welding speed is 11 mm/s, the bottom fusion depth of the weld seam decreases from 2.4 mm to 1.6 mm, and the welding width increases from 12.5 mm to 13.8 mm with the increase in spacing. Furthermore, at a welding speed of 8 mm/s, the lack of fusion at the bottom corner of the narrow-gap groove still exists, but they are not present when welding at a speed of 11 mm/s. It is worth noting that the weld bead exhibits a concave shape under the influence of the narrow-gap groove and the double-wire arc, which is a desired result in narrow-gap welding [34].

**Figure 14:**
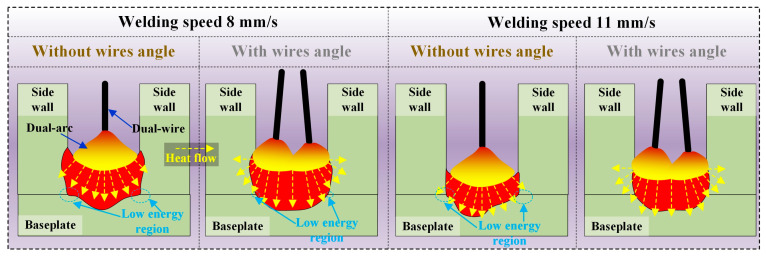
The influence of welding speed and double-wire lateral spacing on weld formation.

The effects of different heat inputs and lateral spacing between the two wires on weld seam formation are illustrated in Figure 14. With a higher heat input, there is more weld metal deposition and a greater filling height, but the high heat of the arc and the molten pool is mainly concentrated on the upper part of the weld seam, making it difficult for the liquid metal to effectively melt the bottom corner of the groove root. With a lower heat input, the weld metal deposition is lower, and the heat from the arc and molten pool can melt the bottom corner of the groove. When there is no lateral spacing between the two wires, the heat from the arc and molten pool is mainly focused on the bottom of the weld seam, making it difficult for the liquid metal to flow to the bottom corner of the narrow-gap groove and achieve metallurgical bonding. When the two wires form a certain angle with each other, the heat from the arc and molten pool can effectively act on the sidewalls, resulting in an increase in welding width and a decrease in bottom fusion depth. Under lower heat input conditions, in particular, the increase in welding width can effectively prevent the occurrence of the lack of fusion during welding.

**Figure 15:**
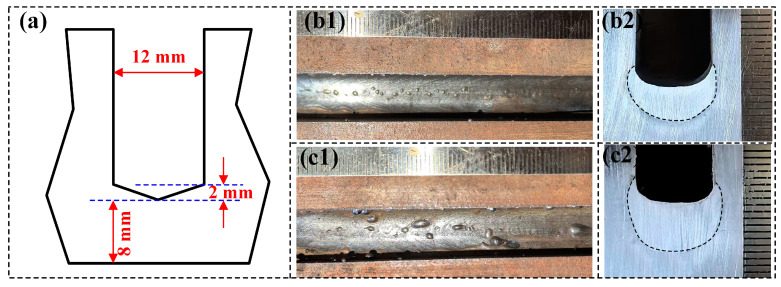
(**a**) The size of the narrow-gap groove and the weld bead formation and cross-sectional morphology at the welding speed of (**b1**,**b2**) 11 mm/s and (**c1**,**c2**) 8 mm/s.

In the case of a type I groove, a larger filling height (about 5 mm) leads to the occurrence of the defect of lack of fusion at the root corner, which may result from the limitation of the groove shape. To address this issue, the groove was machined to have a triangular bottom, as shown in Figure 15a, with the bottom corner of the groove raised 2 mm above the lowest point of the groove, and the width of the gap was 12 mm. Single-pass filling was conducted at the welding speed of 11 mm/s and 8 mm/s using the optimized welding parameters, wire feeding speed of 8 m/min, and lateral spacing of 6 mm at the ends of the two wires. The resulting weld seam formation and cross-sectional morphology are shown in Figure 15b1–c2. It can be observed that under this groove shape, the weld seams are well formed. The weld seams obtained under both higher and lower heat input conditions are concave weld seams. It is worth noting that the lack of fusion at the root corner did not occur, which is attributed to the raised bottom corner of the groove, eliminating the issue of insufficient heat for melting.

### 3.3. Microstructure and Microhardness

**Figure 16:**
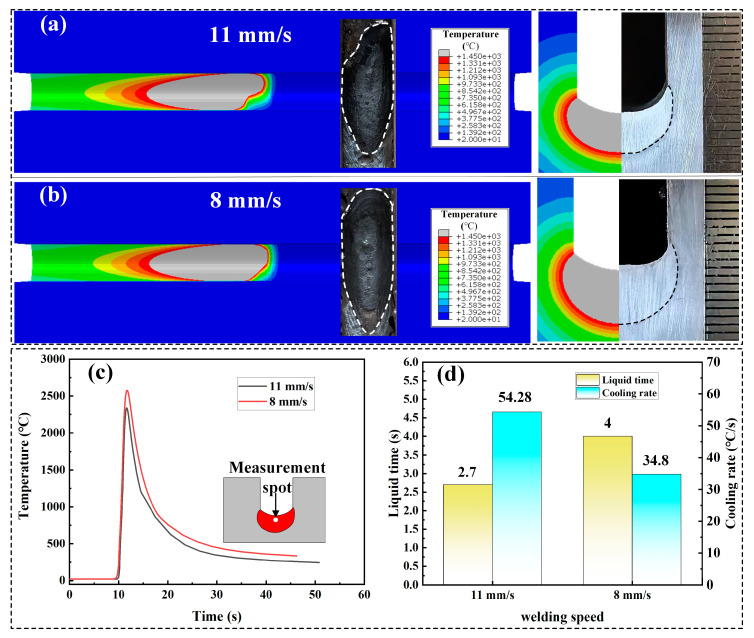
Temperature field simulation results: (**a**,**b**) temperature field contour maps and heat source verification, (**c**) thermal cycle curve at the center of the weld seam, and (**d**) liquid time and cooling rate of the weld seam.

In order to further investigate the heat transfer behavior in narrow-gap welding processes, numerical simulation analysis of the single-pass filling process was conducted using ABAQUS software. Double-ellipsoid heat source is the commonly used equivalent heat source for simulating GMAW processes [35,36]. In this study, a hybrid double–double ellipsoid was used as the heat source for double-wire narrow-gap welding. Figure 16a,b show the temperature field distribution contour maps during single-pass filling welding with welding speeds of 11 mm/s and 8 mm/s, respectively. The gray areas represent temperatures exceeding 1450 °C, approximating the molten pool during the welding process. Both from the top view and cross-sectional view, the simulation results demonstrate a sound match between the equivalent heat source and the actual results, clearly illustrating the temperature field distribution during the welding process. Figure 16c depicts the welding thermal cycle curve at the center of the weld seam for welding speeds of 11 mm/s and 8 mm/s. It can be observed that the peak temperature at the center of the weld seam is higher for a welding speed of 8 mm/s compared to 11 mm/s. Moreover, a higher heat input may result in the filling metal remaining in a liquid state for a longer time and a slower cooling rate for the weld seam. The temperature field simulation results indicate that at a welding speed of 8 mm/s, the existence time of the liquid molten pool is 4 s, which is greater than the 2.7 s at a welding speed of 11 mm/s. Furthermore, at a welding speed of 8 mm/s, the cooling rate of the weld seam is 34.8 °C/s, which is lower than the 54.28 °C/s cooling rate at a welding speed of 11 mm/s.

**Figure 17:**
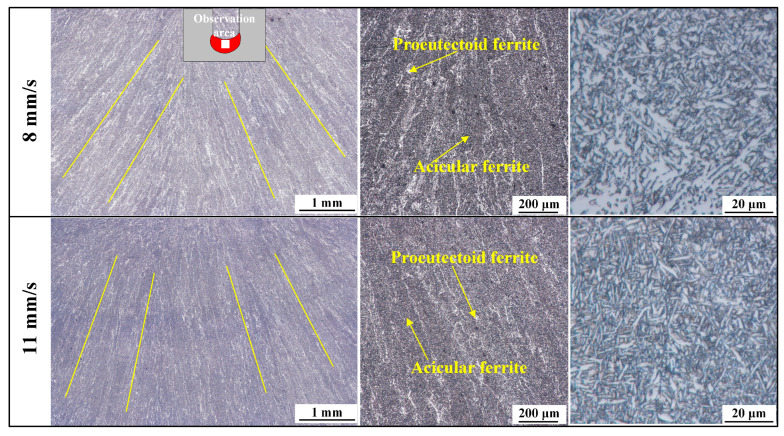
Comparison of the microstructure of welded joints fusion zone at different welding speeds.

Figure 17 shows the microstructure of the fusion zone of welded joints obtained at welding speeds of 8 mm/s and 11 mm/s. The optical micrographs clearly display that the microstructure of the fusion zone exhibits a distinct growth direction, which grows from areas near the base metal toward the top of the weld seam at a certain angle, related to the heat dissipation direction of the molten pool. The microstructure of the fusion zone consists of proeutectoid ferrite (the white areas in the photos) and acicular ferrite (the black areas in the photos). Proeutectoid ferrite is the first to precipitate from the original austenite grain boundaries during the cooling process, while acicular ferrite appears as fine, directional ferrite within the original austenite grains [37,38]. The mechanism of acicular ferrite nuclear is complicated, and their nucleation and growth are affected by the chemical composition, the grain size of austenite, the size and distribution of nucleating particles, and the cooling rate [39,40,41,42]. At a welding speed of 8 mm/s, the content of proeutectoid ferrite in the fusion zone is higher than that at a welding speed of 11 mm/s, whereas the content of acicular ferrite is lower at 11 mm/s. This may be related to the time the liquid phase exists in the fusion zone and the cooling rate of the austenite because of the consistency of the other processing conditions. Numerical simulation results indicate that, at higher welding speed (11 mm/min), the peak temperature of the molten pool is lower (about 2337 °C), the existence time of the liquid metal is shorter, and the cooling rate of the austenite is higher, which promotes the nucleation of acicular ferrite and suppresses the formation of proeutectoid ferrite at high temperatures. High-magnification metallographic images show that the microstructure of the fusion zone of the welded joint obtained at 11 mm/s is denser, which is also related to the faster cooling rate. Similar results were also found in the studies of Lv et al. [43] and Fang et al. [44].

**Figure 18:**
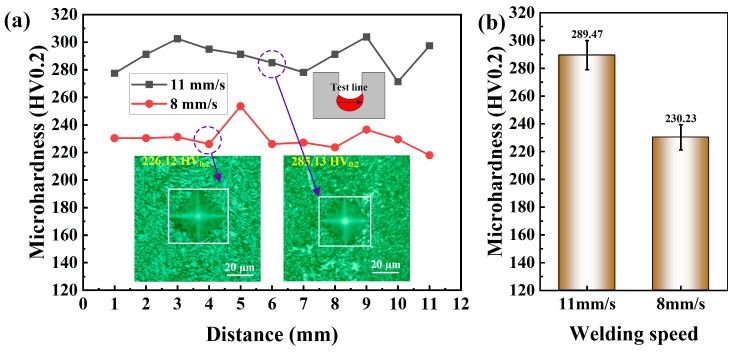
Comparison of microhardness of welded joints fusion zone at different welding speeds: (**a**) microhardness distribution and (**b**) average microhardness value.

Figure 18 compares the microhardness of the fusion zone at welding speeds of 11 mm/s and 8 mm/s. Figure 18a shows the distribution of microhardness in the fusion zone at the two welding speeds. The small fluctuations may be related to differences in the microstructure at different locations. However, it is clear that the microhardness at a welding speed of 11 mm/s is higher than that of 8 mm/s, which is related to the differences in microstructure at the two welding speeds. The inset in Figure 18a provides a comparison of the typical microstructures and diamond indentation impressions at the two welding speeds. It can be seen that the diamond indentation at 11 mm/s is smaller than that at 8 mm/s, and the microstructure near the indentation is notably finer. The higher microhardness value is caused by the finer microstructure. Figure 18b displays the average microhardness of the fusion zone at welding speeds of 11 mm/s and 8 mm/s, which are 289.47 ± 10.47 HV0.2 and 230.23 ± 9.07 HV0.2, respectively.

## 4. Conclusions

This research delves into the application of double-wire narrow-gap gas metal arc welding in low-alloy steel welding. A comprehensive study was conducted on the welding process, weld formation, microstructure, and microhardness of the double-wire narrow-gap GMAW, and a numerical simulation analysis of the welding process was performed. The following conclusions can be drawn:

(1) With the increase in the wire feeding speed from 5 m/min to 8 m/min, the welding current and voltage increase, and the welding arc becomes more and more expanded and more and more bright. The droplet transition mode changes from short-circuit transition mode to spray transition mode.
(2) The gradual increase in welding speed from 5 mm/s to 14 mm/s leads to a reduction in metal filling height and welding width. Lower welding speed leads to undercuts, and higher welding speed leads to a lack of fusion.
(3) The gradual increase in double-wire lateral spacing from 0 mm to 8 mm leads to a larger arc area in the narrow-gap groove, improving the weld formation from finger-like to bowl-shaped. However, the larger lateral spacing of 8 mm would cause the arc to fully burn the sidewall, resulting in undercuts.
(4) When the welding speed is 11 mm/s, the existence time of liquid metal is short, and the cooling rate is fast. This results in a finer acicular ferrite in the weld zone than that of 8 mm/s, which in turn results in a higher microhardness value at a welding speed of 11 mm/s (289.47 ± 10.47 HV0.2) than that of 8 mm/s (230.23 ± 9.07 HV0.2).

## Figures and Tables

**Table 1:** Chemical composition of materials used in this work (wt.%).

Materials	C	Si	Mn	Cr	Ni	Mo	Fe
ER70-G	0.10	0.30	1.45	0.3	1.7	0.35	Bal.
Q235	0.22	0.23	0.56	-	-	-	Bal.

**Table 2:** Parameters for the single-wire welding process.

Group	Welding Speed (mm/s)	Wire Feeding Speed (m/min)	Wire Extension (mm)
1	8	5	25
2		6	
3		7	
4		8	

**Table 3:** Parameters for the double-wire welding process.

Group	Welding Speed (mm/s)		Double-Wire Lateral Spacing (mm)	Wire Extension (mm)
1	5		0	25
2	8			
3	11			
4	14			
5	8	11	2	
6			4	
7			6	
8			8	

## Data Availability

The original contributions presented in the study are included in the article, further inquiries can be directed to the corresponding authors.
